# Broadband Lumped-Element Parameter Extraction Method of Two-Port 3D MEMS In-Chip Solenoid Inductors Based on a Physics-Based Equivalent Circuit Model

**DOI:** 10.3390/mi11090836

**Published:** 2020-09-03

**Authors:** Jiamian Sun, Haiwang Li, Sifan Wu, Tiantong Xu, Hanqing Li, Hanxiao Wu, Shuangzhi Xia

**Affiliations:** 1 School of Energy and Power Engineering, Beihang University, Beijing 100191, China; sunjiamian@buaa.edu.cn (J.S.); 09620@buaa.edu.cn (H.L.); wsf1304@163.com (S.W.); wuhanxiao7652@buaa.edu.cn (H.W.); 2 National Key Laboratory of Science and Technology on Aero-Engine Aero-thermodynamics, Beihang University, Beijing 100191, China; 3 Research Institute of Aero-Engine, Beihang University, Beijing 100191, China; 4 Microsystems Technology Laboratories, Massachusetts Institute of Technology, Boston, MA 02139, USA; hqli@mit.edu; 5 New Era Engineering Consulting Co., Ltd., Beijing 100088, China; shuangzhi_xia@163.com

**Keywords:** lumped-element parameter extraction, microelectromechanical systems (MEMS), in-chip solenoid inductor, physics-based equivalent circuit model

## Abstract

Integrated 2D spiral inductors possess low inductance per unit area, which limits their application range. However, the state of investigation into the lumped-element parameter extraction method for integrated 3D in-chip multi-turn solenoid inductors, which possess higher inductance per unit area, is inadequate. This type of inductor can thus not be incorporated into fast computer-aided design (CAD)-assisted circuit design. In this study, we propose a broadband two-port physics-based equivalent circuit model for 3D microelectromechanical system (MEMS) in-chip solenoid inductors that are embedded in silicon substrates. The circuit model was composed of lumped elements with specific physical meanings and incorporated complicated parasitics resulting from eddy currents, skin effects, and proximity effects. Based on this model, we presented a lumped-element parameter extraction method using the electronic design automation software package, Agilent Advanced Design System (ADS). This method proved to be consistent with the results of two-port testing at low to self-resonant frequencies and could thus be used in CAD-assisted circuit design. The lumped element value variations were analyzed based on the physical meaning of the elements with respect to variations in structures and the substrate resistivity of inductors. This provided a novel perspective in terms of the design of integrated in-chip solenoid inductors.

## 1. Introduction

The inductor is one of the most essential passive components of electronic devices. With trend of miniaturization and integration of electronic devices, increasing attention has been focused on integrated inductors rather than discrete inductors. Integrated inductors are widely used in integrated circuits (IC) and micro electromechanical systems (MEMS) such as Radio Frequency (RF) MEMS [1], microsensors [2], microactuators [3], micro transformers [4], micromotors [5] and micro energy harvesters [6].

Many studies have reported integrated 2D planar inductors, such as meander inductors and spiral inductors, and proposed effective fabrication and packaging solutions which are compatible with complementary metal–oxide–semiconductor (CMOS) processes. However, planar inductors have a lower inductance per unit area compared to solenoid inductors and cannot incorporate magnetic cores. As a result, integrated solenoid inductors are generally considered to be more promising when higher inductance per area is required.

Some researchers have reported integrated solenoid inductors [7,8,9,10,11], but focused on fabrication, analytical models, testing results and all-wave electromagnetic (EM) simulations, rather than on the equivalent circuit model and lumped-element parameter extraction. However, lumped-element equivalent circuit (LEEC) models, also called partial-element equivalent circuit (PEEC) models, and lumped-element parameters are necessary for computer-aided design (CAD)-assisted circuit design, and hence should not be neglected. In circuit simulators, the LEEC models of all components are assembled into a whole circuit, allowing the performance of the whole circuit to be quickly predicted. The analysis of reasonable LEEC models and accurate extracted parameters can indicate the optimization principles for structural design and material selection, especially for frequency-dependent components such as inductors, whose performance is strongly related to parasitics.

While many studies have been performed on circuit models of on-chip spiral inductors [12,13,14,15,16,17,18,19,20], few have investigated models of integrated solenoid inductors. Shin et al. presented LEEC models and parameter extraction for solenoid inductors and successfully predicted the performance of similarly structured inductors [21]. However, the inductors, which were fabricated using a low-temperature co-fired ceramic (LTCC) process, were off-chip components embedded in ceramic materials. Gao et al. reported an on-chip solenoid inductor with a magnetic core and mentioned a three-element compact equivalent circuit model comprised by only a resistor, an inductor and a capacitor but they did not provide deeper analysis of parasitic capacitance [22]. Duplessis et al. presented testing and physics-based theoretical results for the performance of a solenoid inductor and then deduced an equivalent circuit model for a 1-turn solenoid inductor [23]. Based on this model, they extracted parameters using the electronic design automation software package, Agilent Advanced Design System (ADS), but large discrepancies between the test-based and model-based inductance results were recorded about the self-resonant frequency. Chen et al. reported a physics-aware methodology to extract the equivalent circuit model for through-silicon vias (TSV)-inductors [24]. A comprehensive design methodology was proposed involving the initial establishment of a template model. This was followed by the application of initial pole selection and weighting strategies to efficiently find poles and residuals. Finally, an equivalent circuit was extracted using vector fitting. This is currently the most accurate reported methodology. However, the method was complicated, and the final equivalent circuit model comprised many elements that could not be explained theoretically.

As discussed above, although inductor lumped-element parameter extraction is important for circuit design, studies on integrated solenoid inductors have not presented an accurate and concise lumped-element parameter extraction method. In this study, we present a two-port physics-based equivalent circuit model of 3D in-chip solenoid inductors and a broadband lumped-element parameter extraction method based on this model. This method proved to be consistent with two-port testing results at frequencies that varied from very low to self-resonant. This will promote fast CAD-assisted circuit design for 3D in-chip solenoid inductors. We briefly presented some of the results from this study in our previous publication on design, fabrication, modeling and testing of 3D MEMS in-Chip solenoid inductors [25].

## 2. Construction of the Lumped-Element Equivalent Circuit Model

### 2.1. Description of Inductor Structure

**Figure 1:**
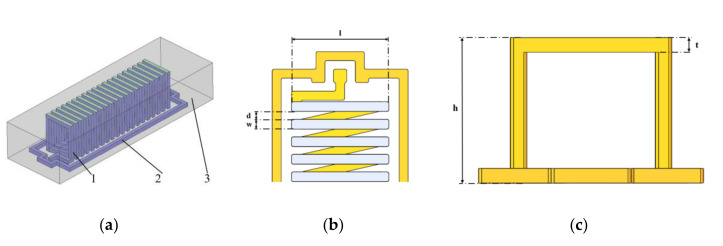
Structure of the inductor. (**a**) Overall view. 1: Inductor conductors; 2: GSG conductors; 3: Silicon substrate. (**b**) Top view without the substrate. l: width of the inductor; d: spacing between adjacent turns; w: linewidth of conductors. (**c**) Front view without the substrate. h: height of the whole inductor; t: height of horizontal conductors (identical to linewidth by default).

To construct the LEEC model of an integrated solenoid inductor, the inductor structure should first be defined. As shown in Figure 1, the 3D multi-turn in-chip solenoid inductor is completely embedded in the silicon substrate. An SiO2 layer is created as the insulation layer using silicon thermal oxidation between the silicon substrate and copper conductors. Ground–signal–ground (GSG) structures surrounding the inductor are necessary for signal shielding in high-frequency testing.

**Figure 2:**
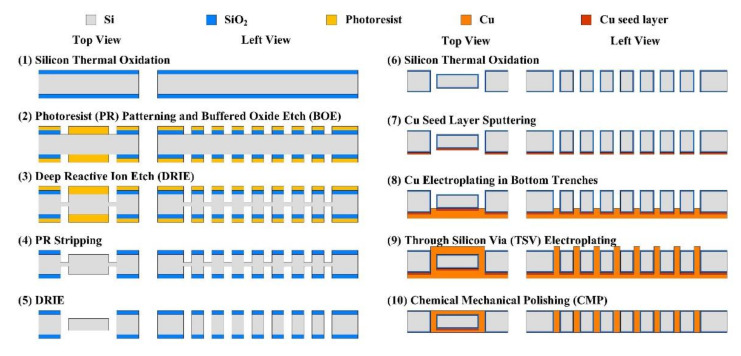
MEMS fabrication process of the inductors.

Various inductor designs were fabricated with good structural integrity and repeatability via a CMOS-compatible MEMS fabrication process, shown in Figure 2. Please refer to our previous work in Ref. [25] for more fabrication details.

### 2.2. Classic Physics-Based LEEC Models

In physics-based LEEC models, all elements have definite physical meanings, although some assumptions are made to simplify the model. These models are more compatible with physical phenomena and theoretical analysis compared to some complex circuit models extracted using numerical fitting methods, such as the method from [24].

**Figure 3:**
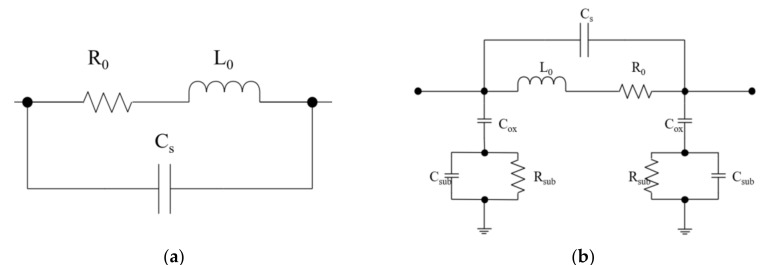
Classic physics-based LEEC models of inductors. (**a**) Three-element compact physics-based LEEC model. (**b**) Single-π LEEC model.

The most compact physics-based LEEC model of an inductor [26] is shown in Figure 3a. In this model, R_0_ and L_0_ are the series electrical resistance and inductance, respectively. The distributed parasitic capacitance is represented by a lumped capacitance connected between the two terminals of the winding. This model is generic for all kinds of inductors but neglects the contribution of the substrate and simplifies all types of parasitic capacitance into one element. Therefore, it is not used as an accurate broadband model.

Another classic physics-based LEEC model is the single-π model [27] shown in Figure 3b. This model was constructed for on-chip spiral inductors, and many subsequent works have proposed improvements to this model. It increases the model accuracy by distinguishing different kinds of parasitic capacitance and adding some resistive and capacitive (RC) elements representing insulating layers and substrates. In addition to the series inductance, *L*_s_, and series resistance, *R*_s_, the capacitive coupling between the two terminals is represented by the series capacitance C_s_. The capacitance between the conductors and insulating oxide is modeled by *C*_ox_. The capacitance and resistance of the substrate are modeled by *C*_sub_ and *R*_sub_.

### 2.3. Physics-Based LEEC Models for In-Chip Solenoid Inductors

In-chip solenoid inductors differ from on-chip spiral inductors in three ways:

(1) On-chip spiral inductors are normally composed of conductors whose bottom surfaces are on the chip surface. Parasitic capacitance thus exists in the air gap between adjacent turns. However, in-chip solenoid inductors are tightly embedded in the substrates. As a result, instead of air, two oxide layers and silicon substrate occupy the space between any two conductors of the solenoid inductor. This means that the parasitic capacitance is composed of two oxide capacitance components and one substrate capacitance component. 

(2) Based on magnetic circuit analysis, more magnetic flux flows through the substrate of solenoid inductors than spiral inductors, which leads to more eddy-current loss in the substrate. Although the single-π model incorporates substrate inductance and electrical resistance, the resistance of the silicon substrate between adjacent turns is not taken into consideration. This cannot be neglected for in-chip solenoid inductors.

(3) Vertical conductors of in-chip solenoid inductors are commonly fabricated using high aspect ratio electroplating of TSVs. The linewidth of the solenoid inductors is much larger than that of the spiral inductors due to electroplating limitations. Therefore, solenoid inductors are influenced more by the skin effect and the proximity effect at high frequencies than spiral inductors.

**Figure 4:**
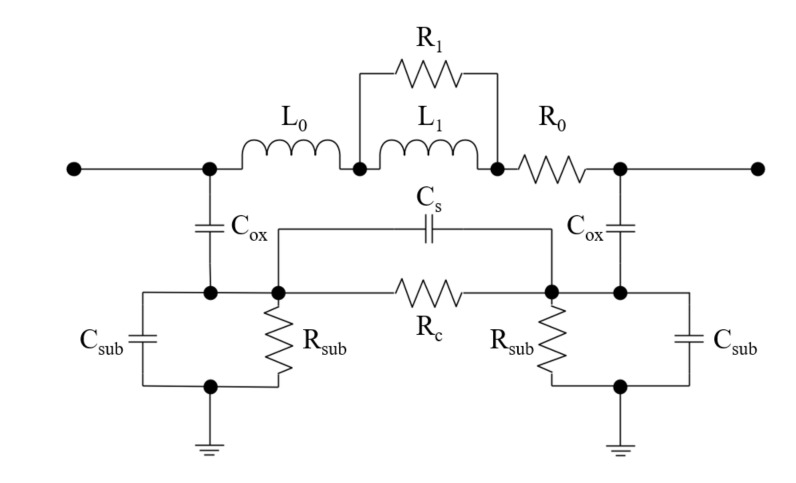
Physics-based LEEC models for in-chip solenoid inductors.

Based on the above analysis, we modified the single-π model in the following ways to construct a new physics-based LEEC model (shown in Figure 4) for in-chip solenoid inductors:

(1) The parasitic capacitance element (*C*_s_) was removed from between the two terminals in the single-pi model. An element (*C*_s_) was added between the two oxide capacitances (*C*_ox_) in the new model to represent the capacitance of the substrate between adjacent turns.

(2) An element (*R*_c_) was added between the two oxide capacitances (*C*_ox_) in the new model to represent the resistance of the substrate between adjacent turns.

(3) Two elements were added in parallel (*R*_1_ and *L*_1_) to the basic series resistance and inductance (*R*_0_ and *L*_0_) to represent the extra electrical resistance and inductance caused by the skin effect and the proximity effect.

This model was constructed based on the real structure of in-chip solenoid inductors, and thus, all the lumped elements have definite physical meanings. It incorporates parasitics, the eddy current effect, the skin effect, and the proximity effect based on the classic models. It has the potential to increase the accuracy and convergence of the following lumped-element extraction method.

## 3. Lumped-Element Parameter Extraction Method

The objective of lumped-element parameter extraction is to determine the values of all LEEC elements that fit a given scattering parameter (S-parameter) dataset of the inductor. The extraction method is divided into two parts: obtaining S-parameter dataset and fitting the LEEC model to the S-parameter dataset.

### 3.1. S-Parameter Dataset Acquisition Method

There are two ways to obtain the S-parameter dataset: testing and EM simulation.

Testing results are considered to be true if the testing is performed correctly. However, it requires significant time and expense to fabricate and test inductor samples of varying geometry, turns, and substrate properties. In addition, testing samples may have undetectable interior defects that result in an unpredictable influence on the testing data. All-wave EM simulation saves lots of time and expense but simulation results without proper verification are not as convincing as testing results. Therefore, obtaining S-parameter datasets by EM simulation is a reasonable way to save time and expenses if the simulation results are verified as accurate by comparison with testing results. 

#### 3.1.1. Testing Method

**Figure 5:**
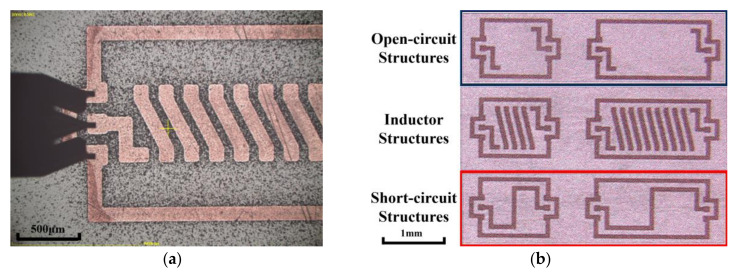
Testing samples. (**a**) Connection of a probe to an inductor embedded in silicon substrates. (**b**) Open-circuit and short-circuit structures beside inductor structures.

To test the performance of sample inductors, a two-port calibrated Agilent N5290A Vector Network Analyzer (VNA) was used. The two test probes were Cascade ACP40-GSG-250 Air Coplanar Probes used in conjunction with the Cascade Summit 12K Probe Station. The three jaws of one test probe were connected to the two ground pads and one signal pad of the inductor terminal (shown in Figure 5a).

In addition to the VNA calibration of the reference samples, extra calibration was done by collecting the testing results from open-circuit and short-circuit structures (shown in Figure 5b). These were fabricated in the same substrate and incorporated the same GSG structures as used in the inductor structures. By proper data processing, the impact of the GSG structures can be eliminated.

The testing results from every sample were collected in an S-parameter dataset over a broad frequency range (1 MHz–100 MHz, interval: 0.05 MHz). The S-parameters had four components denoted as *S*_11_, *S*_12_, *S*_21_, and *S*_22_.

The most important and straightforward parameters for inductor performance evaluation are inductance (*L*), quality factor (*Q*), and resistance (*R*). These can be derived from S-parameter datasets. For a particular frequency, *S*-parameters can be converted to admittance parameters (*Y*-parameters) as follows:

(1) $$Y_{11}=\frac{1-S_{11}+S_{22}-S_{11}\cdot S_{22}+S_{12}\cdot S_{21}}{1+S_{11}+S_{22}+S_{11}\cdot S_{22}-S_{12}\cdot S_{21}},$$

The *Y*-parameters can then be converted to impedance parameters (*Z*-parameters) as follows:

(2) $$Z_{11}=\frac{1}{Y_{11}},$$

Finally, the inductance, quality factor, and resistance can be obtained as follows:

(3) $$L=\frac{imag(Z_{11})}{2\times \pi \times f},\;Q=\frac{imag(Z_{11})}{real(Z_{11})},\;R=real(Z_{11})$$

#### 3.1.2. Accuracy Verification of EM Simulation Method

**Figure 6:**
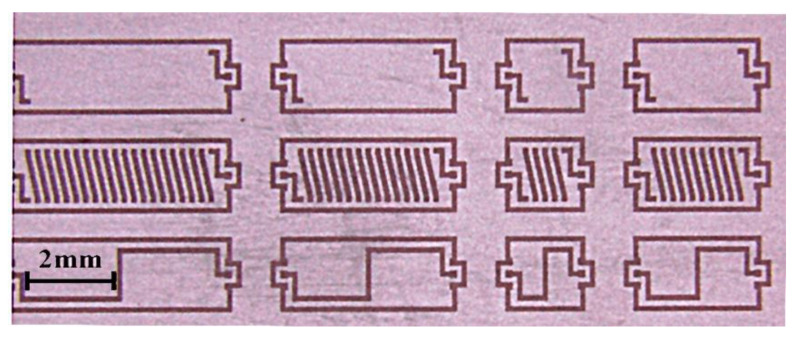
Four tested samples embedded in a silicon wafer.

To simulate the performance of the inductors, an all-wave EM field-simulating software package, ANSYS high-frequency structure simulator (HFSS), was used. To verify the EM simulation accuracy, the geometry and material properties of the simulated objects (shown in Table 1 and Figure 6) were made identical to the four tested samples, and the excitation source was set to lumped port mode to correspond with the testing method.

**Figure 7:**
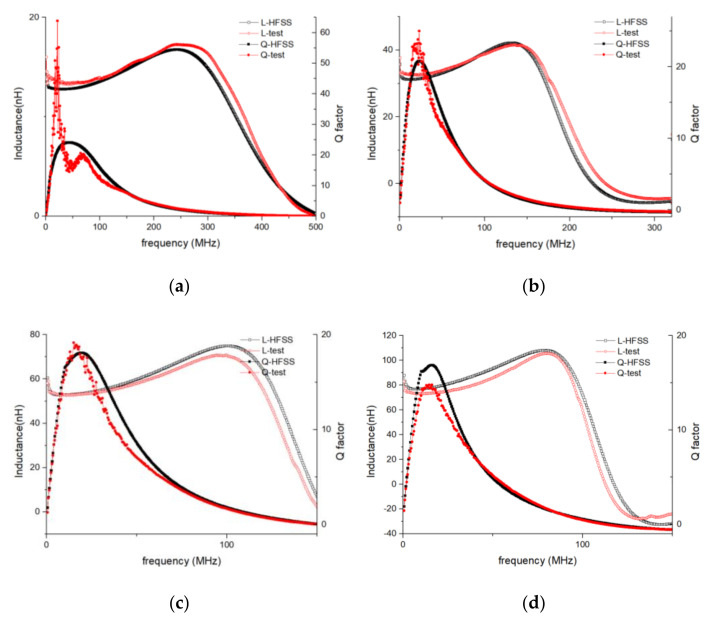
EM-simulation and test *L*/*Q* vs. frequency curves of variable-turn inductors. L-HFSS and *Q*-HFSS are plots of the EM results simulated using ANSYS HFSS. The *L*-test and *Q*-test are plots of the test results. (**a**) *N* = 5, (**b**) *N* = 10, (**c**) *N* = 15, and (**d**) *N* = 20.

The accuracy of the EM simulation method was then verified by comparing the simulated and tested *L*/*Q* vs. frequency curves (shown in Figure 7). Good agreement can be seen between the simulation and test curves over a broad frequency range. The largest errors were recorded for the *Q*-peak frequency, where the test curves fluctuated wildly. It is reasonable to expect that the errors in the *Q* vs. frequency curves would be larger than those in the *L* vs. frequency curves, because fabrication defects and errors have a more significant effect on *Q* than on *L*.

The EM simulation accuracy was thus verified as acceptable. Therefore, the simulation-based *S*-parameter datasets can be used as fitting targets for LEEC parameter extraction.

### 3.2. LEEC Model Fitting Method

**Figure 8:**
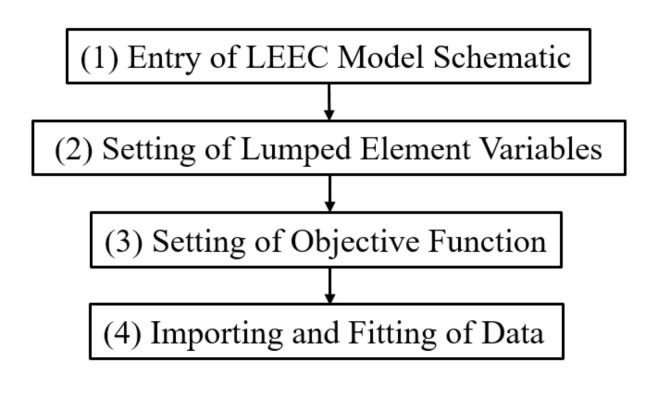
LEEC model fitting flowchart.

To fit the LEEC model to an S-parameter dataset for a particular in-chip solenoid inductor, frequency-domain circuit simulation was performed using an electronic design automation software package, ADS. The model fitting process is shown in Figure 8 as follows:

The first step is the LEEC model schematic entry. The model constructed in this study was entered into the ADS.

The second step is the lumped element variable setting. The variables are the values of the lumped elements, which were determined by using the following fitting iteration. As shown in Figure 4, the circuit was composed of 11 elements, among which *C*_s_, *R*_sub_, and *C*_ox_ appeared twice, respectively. The values of the eight different elements were set as eight variables to be fitted. The initial variable values and their range in the following iteration were then defined reasonably to avoid non-convergence, slow convergence, and multiple solutions by theoretical calculation as presented in references [28,29,30]. Based on a group of variable values, the performance parameters could be obtained by circuit simulation performed using ADS.

The third step was the setting of the objective function. 

Objective functions indicate the error between the given EM-simulation-based data and the circuit-simulation-based fitted data. Errors between the given data and the fitted data for the six performance parameters at three critical frequency points were defined as six objective functions. 

Generally, *S*-parameters, Y-parameters, inductance (*L*), and quality factor (*Q*) are all performance parameters that can be selected as compared parameters in objective functions. Among the four components of the Y-parameters for inductors, which are symmetrical passive two-port networks, *Y*_11_ = *Y*_22_, *Y*_12_ = *Y*_21_. Because *S*-parameters can be converted into Y-parameters, the amplitudes and phases of *Y*_11_ and *Y*_12_, along with *L* and *Q*, can be selected as the six compared parameters. These six objective functions proved to be a good selection in terms of the fitting accuracy and the comparative time consumed over a large number of fitting experiments.

The three critical frequency points were the test starting frequency (1 MHz in this study), the *Q*-peak frequency, and the self-resonant frequency (SRF).

The last step was data importing and fitting. The steps in this process were as follows:

(1) The EM-simulation-based S-parameter dataset was imported into ADS and converted to obtain EM-simulation-based data for the six performance parameters. 

(2) The LEEC was simulated using initial values of variables to obtain circuit-simulation-based data for the six performance parameters.

(3) The values of the six objective functions were calculated by substituting the EM-simulation-based and circuit-simulation-based data into objective functions.

(4) A convergence tolerance was set. If all the values of the objective functions were not smaller than the tolerance, a fitting iterative algorithm provided by ADS was selected and iteration was performed using the variables until all the function values were under tolerance. The final group of variables as determined by the lumped element parameters was then output.

## 4. Results and Discussion

### 4.1. Lumped-Element Parameter Extraction Method Accuracy Verification

**Figure 9:**
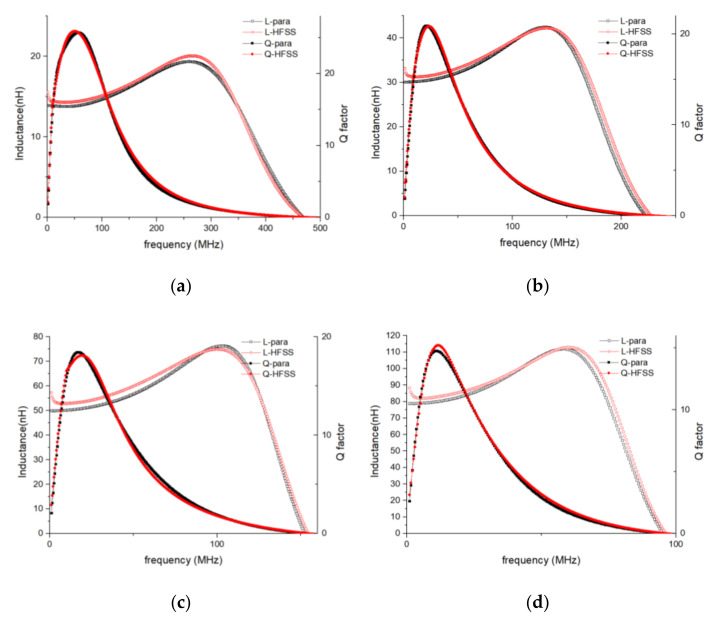
EM-simulation-based and extracted-parameter-based *L*/*Q* vs. frequency curves of variable- turn inductors. *L*-HFSS and *Q*-HFSS are plots of the EM-simulated results from ANSYS HFSS. *L*-para and *Q*-para are plots of the simulation-based results of a circuit incorporating the parameters extracted from ADS. (**a**) *N*=5, (**b**) *N* = 10, (**c**) *N* = 15, and (**d**) *N* = 20.

The element values of four inductors (the four inductors shown in Table 1) were extracted in accordance with the LEEC model and lumped-element parameter extraction method. Based on the extracted values, the performance data were then simulated and compared with the data converted from the given EM-simulation-based S-parameter datasets (shown in Figure 9).

The comparison shows that the *L* and *Q* of the extracted-parameter-based results were in good agreement with the EM-simulation-based results. Although there were relatively large errors in the *Q* vs. frequency curves at frequencies under 5 MHz, very good agreement with only small associated error (< 5%) can be seen in other parts of the curves. Considering that the in-chip inductor normally functions at frequencies about the *Q*-peak frequency, these errors were acceptable.

This lumped parameter extraction method balances simplicity and accuracy, and thus provides acceptable accuracy with low time consumption. Therefore, it was deemed to be a good method to quickly characterize an in-chip solenoid inductor and incorporate the inductor into a fast CAD-assisted circuit design.

### 4.2. Influence of Inductor Geometry and Substrate Resistivity on Extracted Parameters

As described in Section 2, the constructed LEEC model is physics-based, and thus all the elements have specific physical meanings. In brief, *L*_0_ and *R*_0_ represent direct current inductance and resistance, respectively; *C*_si_ and *C*_ox_ represent the parasitic capacitance produced by electric field coupling between the inductor conductors and substrates; *R*_si_ represents substrate loss caused by the eddy current effect at high frequencies; *L*_1_ and *R*_1_ represent the extra inductance and electrical resistance caused by the skin effect and proximity effect at high frequencies. 

Variation in geometry and substrate resistivity (*N*, *d*, *l*, *s*, and *ρ* shown in Table 1) have a clear influence on inductor performance. Therefore, using the above parameter extraction method, the corresponding influence on lumped-element parameters could be derived to analyze the physical effects and design principles of in-chip solenoid inductors. The detailed analysis is as follows:

(1) Influence of inductor turns (*N*). 

**Figure 10:**
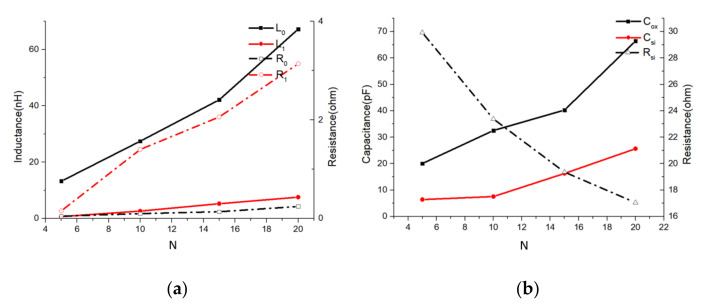
Lumped-element parameters vs. N curves. (*d* = 100 μm; *l* = 1000 μm; *s* = 1 μm) (**a**) *L*_0_/*L*_1_/*R*_0_/*R*_1_ vs. *N* curves. (**b**) *C_ox_/C_si_/R_si_* vs. *N* curves.

According to Figure 10, when the number of inductors increases, *L*_0_, *L*_1,_ *R*_0_, *R*_1_, *C*_si_, and *C*_ox_ show an approximately linear rise, which is as expected. The *R*_si_ decreased, which indicated that the eddy effect became stronger and substrate loss increased.

(2) Influence of spacing between adjacent turns (d).

**Figure 11:**
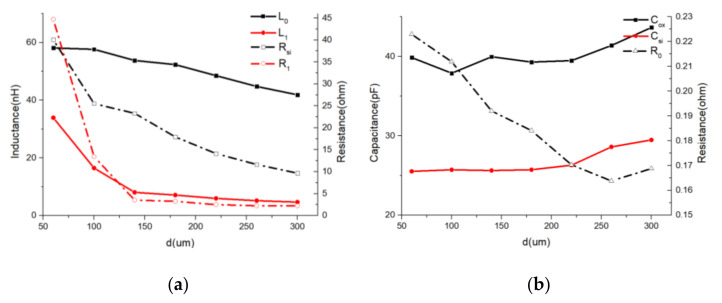
Lumped-element parameters vs. d curves. *(l* = 1000 μm; *s* = 1 μm; *N* = 20) (**a**) *L*_0_/*L*_1_/*R_si_*/*R*_1_ vs. d curves. (**b**) *C_ox_*/*C_si_*/*R*_0_ vs. d curves.

According to Figure 11, when the spacing between adjacent turns increases, *L*_0_ decreases slowly because the magnetic flux leakage increases. *L*_1_ and *R*_1_ decreased rapidly at first and then the declining trend slowed down. This indicated the influence of the proximity effect. The *R*_si_ decreased, which indicated that the substrate loss increased.

(3) Influence of inductor width (l).

**Figure 12:**
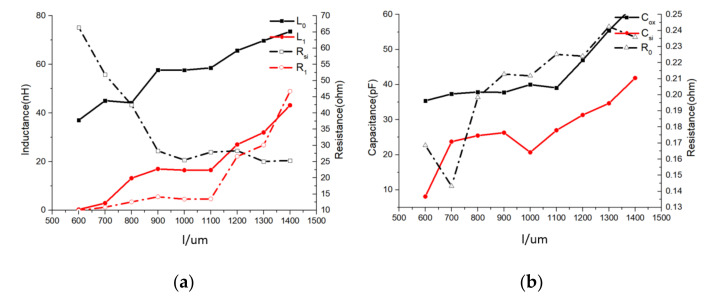
Lumped-element parameters vs. l curves. (*d* = 100 μm; *s* = 1 μm; *N* = 20) (**a**) *L*_0_/*L*_1_/*R_si_*/*R*_1_ vs. l curves. (**b**) *C_ox_*/*C_si_*/*R*_0_ vs. l curves.

According to Figure 12, when the inductor width increases, *L*_0_, *L*_1_, *R*_0_, *C_si_*, and *C*_ox_ show an approximately linear rise, which is as expected. However, R_1_ increased rapidly, indicating that the proximity effect rapidly became stronger. The R_si_ showed a decreasing trend but the rate of change decreased, which indicated a similar increasing trend in substrate loss.

(4) Influence of oxide insulating layer thickness (s).

**Figure 13:**
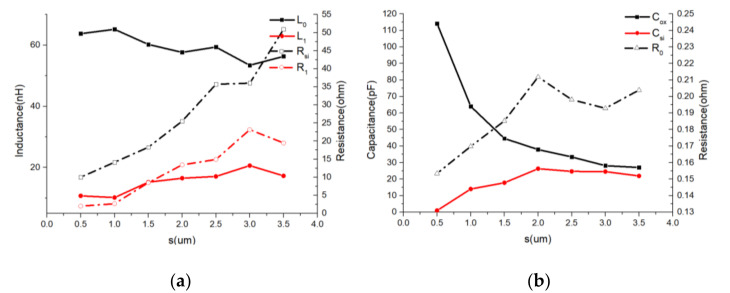
Lumped-element parameters vs. s curves. (*d* = 100 μm; *l* = 1000 μm; *N* = 20) (**a**) *L*_0_/*L*_1_/*Rsi*/*R*_1_ vs. s curves. (**b**) *C_ox_*/*C_si_*/*R*_0_ vs. *s* curves.

According to Figure 13, when the oxide thickness increases, *L*_0_, *L*_1_, *R*_0_, and *R*_1_ show a slowly changing trend. However, the equivalent sum of *C*_si_ and *C*_ox_ decreased significantly and *R*_1_ increased rapidly, which indicated that parasitic capacitance and substrate loss decreased. Therefore, thicker oxides were noted as being beneficial to inductor performance.

(5) Influence of silicon substrate resistivity (ρ).

**Figure 14:**
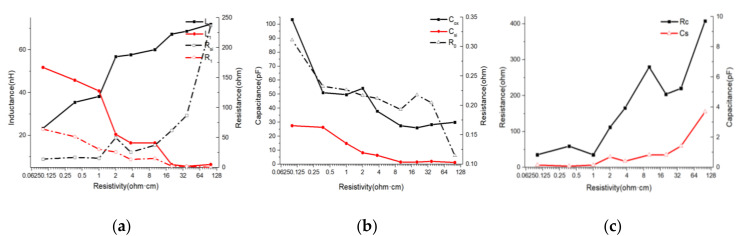
Lumped-element parameters vs. ρ curves. (*d* = 100 μm; *l* = 1000 μm; *N* = 20; *s* = 1 μm) (**a**) *L*_0_/*L*_1_/*Rsi*/*R*_1_ vs. ρcurves. (**b**) *C_ox_/C_si_/R_0_* vs. ρcurves. (**c**) *R_c_/C_s_* vs. ρcurves.

According to Figure 14, when the silicon substrate resistivity increased, the sum of *L*_0_ and *L*_1_, remained approximately constant. However, the sum of *R*_0_ and *R*_1_ showed a rapidly decreasing trend, which indicated an increase in performance. The equivalent sum of *C*_s_, *C*_si_, and *C*_ox_ decreased significantly, which indicated that the parasitic capacitance decreased. The equivalent sum of *R*_si_ and *R*_c_ increased rapidly, which indicated that the substrate loss decreased. Therefore, higher substrate resistivity improved inductor performance.

Based on the above analysis, the inductor performance changes caused by variations of some parameters can be discussed from a novel perspective because the performance changes can be explained with changes in the lumped-element parameters. Although the above lumped element parameter curves showed some fluctuations that were difficult to explain, they did provide new viewpoints in terms of the design of in-chip solenoid inductors.

## 5. Conclusions

In this study, we presented a two-port physics-based equivalent circuit model of a 3D MEMS in-chip solenoid inductor and a broadband lumped-element parameter extraction method based on this model. A frequency-independent physics-based twelve-element LEEC model was proposed for 3D solenoid inductors which are embedded in silicon substrates. The model incorporated the complicated parasitics resulting from the eddy current effect, skin effect, and proximity effect. A lumped-element parameter extraction method was then proposed which incorporated two parts: obtaining the S-parameter dataset and parameter fitting based on the constructed physics-based LEEC model. The results for different inductors modeled in the full-wave EM field tool ANSYS HFSS proved to be accurate compared to the test results. These were then imported into the ADS electronic design automation software package to extract the lumped-element parameters. The extracted parameters were verified as sufficiently accurate, which indicated that the method was effective. The variations in lumped element values were then analyzed with respect to varying geometries and substrate resistivity to develop in-chip solenoid inductor design principles. This method proved to be consistent with two-port testing results at frequencies that varied from low to SRF and thus could be applied to other in-chip solenoid inductors. This will promote fast CAD-assisted circuit design for 3D in-chip solenoid inductors. We plan to apply this methodology to micro transformers with in-chip solenoid coils.

In this study, we balanced complexity and accuracy of this methodology. The accuracy is acceptable for CAD-assisted inductor design while the computing speed is quick thanks to the physics-based eight-variable LEEC. However, the lumped element parameter curves shown in Figure 10, Figure 11, Figure 12, Figure 13 and Figure 14 had some fluctuations that were difficult to explain. This indicates performance of inductors with different geometries cannot be accurately predicted using these curves, which is a limitation for this methodology.

## Figures and Tables

**Table 1:** Geometry and material properties of four simulated and tested inductors.

Symbol	Parameter	Value	Symbol	Parameter	Value
l	inductor width/μm	1000	d	spacing between adjacent turns/μm	100
w	linewidth of conductors/μm	100	h	height of the whole inductor/μm	1000
t	height of horizontal conductors/μm	100	N	inductor turns	5/10/15/20
s	oxide insulating layer thickness/μm	1	ρ	silicon resistivity/(Ω·cm)	3.7

The geometry parameters are marked in Figure 1.
